# Proliferative Tumor States and Immunogenic Ecosystems Predict Neoadjuvant Chemotherapy Response in Triple-Negative Breast Cancer

**DOI:** 10.3390/biomedicines14030643

**Published:** 2026-03-12

**Authors:** Yuan Teng, Huan Li, Lin Cheng, Yingming Jiang, Hua Jiang, Yu Liu

**Affiliations:** 1 Department of Thyroid and Breast Surgery, Third Affiliated Hospital of Sun Yat-Sen University, Guangzhou 510630, China; 2 Department of Breast and Thyroid Surgery, Guangzhou Women and Children’s Medical Center, Guangzhou Medical University, Guangzhou 510370, China; tengyuangz@163.com

**Keywords:** triple-negative breast cancer, neoadjuvant chemotherapy, single-cell RNA sequencing, tumor microenvironment, T cell receptor repertoire, B cell receptor repertoire, glycosylation, immune checkpoints, multi-omics

## Abstract

**Background:** Triple-negative breast cancer lacks established targeted therapies, and only a subset of patients achieves a pathologic complete response to neoadjuvant chemotherapy. We aimed to integrate bulk cohorts with an exploratory single-cell multi-omic dataset from only five patients to identify tumor and immune-related features associated with chemotherapy response. **Methods:** Bulk analyses were performed in two public breast cancer cohorts (GSE76275 and GSE25065) to compare triple-negative versus non-triple-negative tumors and to relate pretreatment transcriptional and inferred immune infiltration patterns to neoadjuvant chemotherapy response. Separately, in a hypothesis-generating single-cell cohort of five triple-negative breast cancers (n = 5; four responders, one non-responder), we performed single-cell RNA sequencing, T cell and B cell receptor sequencing, single-cell ATAC sequencing, and glycosylation tag profiling. **Results:** In bulk data, triple-negative tumors showed a loss of luminal estrogen receptor-associated programs, higher proliferation, and CIBERSORT-estimated enrichment of myeloid-associated immune fractions compared with non-triple-negative tumors. Chemotherapy response was associated with modest transcriptional shifts and inferred immune composition differences in triple-negative tumors and more pronounced epithelial, stromal, and inflamed immune changes in non-triple-negative disease. Single-cell data suggested that responder tumors were enriched for T and natural killer cells, antigen-presenting myeloid cells, expanded and diverse T and B cell clonotypes, and immune-associated glycosylation signals, whereas the non-responder sample was dominated by epithelial and fibroblast compartments with secretory, adhesion, and potential immune evasion programs. Checkpoint-related analyses reflected expression patterns and predicted ligand–receptor communication, nominating TIGIT–NECTIN2 as a candidate axis for further investigation. **Conclusions:** Integrating public bulk cohorts with exploratory single-cell multi-omics supports a model in which chemotherapy sensitivity in triple-negative breast cancer is linked to inflamed, antigen-presenting microenvironments and adaptable antitumor immunity, whereas resistance is associated with stromal and tumor dominance. These candidate biomarkers and pathways require validation in larger independent cohorts, and clinical translation is premature given the exploratory single-cell cohort.

## 1. Introduction

Breast cancer is the most commonly diagnosed malignancy in women and a leading cause of cancer mortality worldwide, with a rising global burden that continues to strain health systems [1]. Triple-negative breast cancer (TNBC), defined by the absence of estrogen receptor, progesterone receptor, and human epidermal growth factor receptor 2, represents approximately 15 to 20 percent of cases and displays clinical and biological heterogeneity that manifests as early-onset, rapid progression, high metastatic potential, and inferior survival compared with other subtypes [2].

In the absence of broadly effective molecular targets, cytotoxic chemotherapy remains the therapeutic backbone for early-stage TNBC. Anthracycline and taxane-containing regimens are standard in the adjuvant and neoadjuvant settings, and neoadjuvant chemotherapy (NAC) is preferred for most stage II–III presentations because it facilitates down-staging, enables response assessment, and informs post-operative management [3]. Pathologic complete response after NAC correlates strongly with improved event-free and overall survival in TNBC, yet only about one third to one half of patients achieve this outcome with chemotherapy alone, underscoring persistent therapeutic resistance in a substantial subset [4,5,6]. In parallel, immunotherapy-containing neoadjuvant regimens have reshaped standard management for high-risk early-stage TNBC, with pembrolizumab plus chemotherapy followed by adjuvant pembrolizumab improving pCR and event-free survival in KEYNOTE-522 and informing contemporary guideline-based recommendations [3,6,7]. Contemporary reviews and guidelines converge on this range and emphasize the need for response-directed strategies [3,8,9].

The biological basis of divergent NAC responses in TNBC is multifactorial. Bulk transcriptomic profiling has delineated disease-level subtypes, but it averages across cellular compartments and can obscure the contribution of rare cell states and stromal or immune niches [10]. Moreover, many bulk predictors reflect composite signals influenced by tumor purity and variable immune or stromal admixture, limiting cellular attribution and mechanistic interpretability. Integrating bulk cohorts with single-cell and spatially resolved multi-omics is therefore expected to improve biological resolution by mapping transcriptional programs to specific malignant and microenvironmental cell states and by adding orthogonal layers such as chromatin accessibility and lymphocyte clonotypes [11,12,13]. In TNBC, longitudinal single-cell DNA and RNA profiling across neoadjuvant chemotherapy indicates that resistant genotypes can be present before treatment and become selectively enriched, while transcriptional resistance programs can emerge through therapy-associated reprogramming, supporting complementary selection and adaptation mechanisms in non-response [14]. Spatially resolved profiling further suggests that therapeutic benefit depends on the tissue organization of immune and tumor states, including the proximity of cytotoxic lymphocytes to antigen-presenting myeloid niches and epithelial programs, and that these architectures can change early on treatment [13,15]. Consistently, spatial transcriptomic and genomic profiling of residual disease after neoadjuvant chemotherapy highlights TNBC-specific immune and macrophage interaction programs associated with clinical outcomes, underscoring that residual ecosystems are not interchangeable across subtypes [14,15]. Together, these TNBC-focused studies motivate integrating single-cell and spatial information with bulk cohorts to nominate mechanistically grounded candidate biomarkers and pathways for subsequent validation.

Hence, we integrate clinical data with single-cell and bulk RNA sequencing to compare NAC responders and non-responders in TNBC. We characterize differences in tumor-intrinsic programs, immune and stromal ecosystems, and putative resistance circuits, and we nominate candidate multi-omic features and interaction axes associated with NAC response for subsequent validation in larger independent cohorts rather than for immediate patient stratification.

## 2. Materials and Methods

### 2.1. Data Acquisition and Candidate DEG Screening

Transcriptional data were collected from the Gene Expression Omnibus (GEO) datasets GSE76275 and GSE25065 [16,17,18]. The GSE76275 dataset consists of 198 TNBC (triple-negative breast cancer) samples and 67 non-TNBC samples, while the GSE25065 dataset includes 64 TNBC and 134 non-TNBC samples. Raw expression data were downloaded at the probe level and converted to gene-level expression using platform-specific annotations (GPL570 for GSE25065 and GPL96 for GSE76275). These cohorts were selected because they provide pretreatment bulk expression with subtype annotation and NAC response labels at a sample size sufficient for within-cohort subtype and within-subtype response contrasts, while avoiding the heterogeneity introduced by pooling NAC datasets that differ in platform, preprocessing, and response definitions.

Differentially expressed genes (DEGs) between TNBC and non-TNBC tumor samples were identified using the limma R package. The selection criteria for DEGs were |log2 fold change (FC)| > 1 and adjusted *p*-value < 0.05. The Benjamini–Hochberg method was applied to control the false discovery rate (FDR) at the level of each contrast. To minimize platform-related batch artifacts, GEO cohorts were analyzed independently and were not merged across platforms; within each cohort, we inspected sample-level expression distributions and unsupervised similarity patterns to exclude gross technical outliers prior to differential expression analyses. In addition to the TNBC vs. non-TNBC comparison, we further stratified the GSE25065 dataset to explore the association between neoadjuvant chemotherapy (NAC) response and gene expression within each subtype. Specifically, we compared TNBC samples with NAC responders vs. non-responders, as well as non-TNBC samples with NAC responders vs. non-responders.

### 2.2. Functional Enrichment Analysis

To investigate the biological processes and pathways associated with the DEGs, we performed Gene Ontology (GO) and Kyoto Encyclopedia of Genes and Genomes (KEGG) pathway enrichment analysis. DEGs identified in the bulk datasets were subjected to functional enrichment using the clusterProfiler and org.Hs.eg.db R packages. Pathways and terms were ranked by adjusted *p*-values, and FDR control was applied within each analysis. The top enriched GO terms and KEGG pathways were visualized as bar plots and dot plots to highlight the most strongly associated biological processes, molecular functions, cellular components and pathways. To reduce redundancy among GO categories and avoid over-interpretation driven by overlapping terms, we applied semantic similarity-based reduction and reported representative non-redundant GO terms in the main text and figures. These results were interpreted as providing context for the transcriptional differences observed rather than as definitive evidence of pathway activation.

### 2.3. Immune Infiltration Analysis

Immune cell infiltration was estimated using the CIBERSORT algorithm, which infers the relative abundance of different immune cell types from bulk gene expression profiles. Immune infiltration analysis was performed separately for the GSE25065 and GSE76275 datasets. For each dataset, we obtained immune cell proportion estimates and compared TNBC versus non-TNBC tumors, and NAC responders versus non-responders within each subtype. Immune cell proportions were summarized using boxplots, and group comparisons were assessed using non-parametric tests. In view of the number of immune subsets tested and the exploratory nature of these analyses, *p*-values were primarily interpreted descriptively; particular emphasis was placed on patterns that were consistent across related cell types and datasets rather than on single nominally significant comparisons. Spearman’s correlation was used to evaluate co-variation among immune cell types, and correlation matrices were visualized as heatmaps to highlight coordinated immune modules. The correlation between selected target genes (identified from DEG analyses) and immune cell types was also examined using Spearman’s correlation and displayed in heatmaps. These analyses were designed to generate hypotheses about immune microenvironmental patterns associated with TNBC and NAC response. CIBERSORT was run in relative mode using the LM22 signature matrix with 1000 permutations, and only samples with CIBERSORT deconvolution *p* < 0.05 were retained for responder versus non-responder comparisons. Because these deconvolution outputs represented estimated immune fractions and multiple immune subsets were tested, we interpreted differences descriptively and emphasized directionally consistent patterns across related immune subsets rather than relying on nominal *p*-values from individual subsets.

### 2.4. Human Sample Collection

This study enrolled five patients with histologically confirmed triple-negative breast cancer who received neoadjuvant chemotherapy at our institution. Responders were defined as achieving pathologic complete response after NAC (ypT0/is and ypN0), whereas the single non-responder had residual invasive disease (no pathologic complete response, non-pCR) after NAC by institutional pathology. Written informed consent was obtained from all participants prior to sampling. The study protocol was approved by the institutional review board (approval number: II2024-215-01).

Inclusion criteria were a clinical history consistent with triple-negative disease with estrogen receptor, progesterone receptor and HER2 all negative on immunohistochemistry, confirmation of breast cancer by at least two imaging modalities (breast ultrasound plus computed tomography or magnetic resonance imaging), and histopathological confirmation on biopsy. Exclusion criteria were documented human immunodeficiency virus infection, a history of another malignant tumor, and current pregnancy or within six months postpartum.

Fresh surgical specimens were obtained intraoperatively after consent. Sampling targeted the primary tumor, metastatic lymph node when available, and para-tumor tissue. In this study, sample denotes an independently processed tissue specimen and its corresponding single-cell library. Immediately after resection, tissue pieces were placed on ice in Singleron sCelLive Tissue Preservation Solution and transferred to the laboratory within thirty minutes. Preoperative clinical data collected included age and sex, core biopsy lesion size and clinical stage, immunohistochemical markers including ER, PR and HER2, complete blood count, lesion size before and after treatment, and the clinical response category. Given the small sample size (n = 5, 4 responders and 1 non-responder), all subsequent single-cell and multi-omic analyses were pre-specified as exploratory and hypothesis-generating.

### 2.5. Wet-Lab Validation and Single-Cell Multi-Omic Profiling

Fresh tumor specimens were washed, mechanically minced and enzymatically dissociated using the Singleron PythoN system^®^, followed by filtration, red blood cell lysis and PBS washes to obtain single-cell suspensions with >85% viability for library preparation. Single-cell RNA-seq libraries were generated on the Singleron Matrix/GEXSCOPE platform according to the manufacturer’s protocol and sequenced on an Illumina NovaSeq 6000 with 150 bp paired-end reads at a target depth of approximately 50,000 reads per cell [19]. In parallel, immunoreceptor profiling was performed using the GEXSCOPE Immuno-TCR/BCR kit to obtain matched scTCR- and scBCR-seq libraries [20], and surface glycosylation was assessed with the ProMoSCOPE kit, in which cell-surface glycans were tagged with fucose-based oligonucleotides and processed through the same workflow before sequencing on NovaSeq [21,22]. Chromatin accessibility was profiled by scATAC-seq: nuclei were gently isolated, processed with the 10× Genomics Chromium Single Cell ATAC solution and sequenced with 150 bp paired-end reads on NovaSeq [23]. For ProMoSCOPE, glyco tag UMI counts were quantified per cell from the same barcoded libraries. For downstream analyses, glyco tag counts were normalized at the single-cell level by scaling each cell to a fixed glyco tag library size of ten thousand followed by log1p transformation. For visualization, normalized tag values were additionally scaled within each cell type. Because baseline tag abundance and capture can vary across lineages, comparisons were interpreted primarily within the same cell type across samples and summarized at the patient level rather than treating each cell as an independent replicate.

For scRNA sequencing and ProMoSCOPE, raw reads were processed with CeleScope v1.15.0. Barcodes and UMIs were extracted from R1 and corrected. R2 was trimmed with Cutadapt v3.7 to remove adapters and poly(A). Trimmed reads were aligned to GRCh38 with STAR v2.6.1b. Uniquely mapped reads were assigned to genes using featureCounts v2.0.1. UMIs were collapsed per cell gene to generate count matrices [24,25,26]. Quality control was performed in Scanpy v1.8.2 [27,28,29]. Cells with fewer than 200 detected genes, the top two percent of gene counts or UMIs, or mitochondrial content greater than fifteen percent were removed. Genes expressed in fewer than five cells were dropped. After filtering, 121,666 cells remained. Counts were normalized to total UMIs per cell and log transformed. The two thousand most variable genes were selected with the Seurat flavor. Principal components were computed on scaled data. The top twenty principal components supported Louvain clustering at resolution 1.2 and UMAP visualization [27,28,29]. Batch effects across patients and tissues were mitigated with Harmony v1.0 using the first twenty principal components [27,28,29]. For scATAC sequencing, nuclei with fewer than one thousand fragments or transcription start site enrichment less than four were removed. Bins overlapping ENCODE blacklist regions were excluded [30,31]. Dimensionality reduction used iterative latent semantic indexing. Clustering used ArchR v1.0.1 with SLM and UMAP for visualization [30,31]. Cell types were annotated using Cell ID against SynEcoSys reference signatures and then verified by canonical marker expression [32]. Major lineages were confirmed using EPCAM, KRT8, KRT18 for epithelial cells; COL1A1, COL1A2, DCN for fibroblasts; PECAM1, VWF for endothelial cells; PTPRC for leukocytes; LST1, TYROBP, C1QA for mononuclear phagocytes; CD3D, CD3E, TRAC for T cells; NKG7, GNLY for NK cells; MS4A1, CD79A, CD74 for B cells; and S100A8, S100A9, FCGR3B for neutrophils. Immune subtypes were cross-checked with established markers, including CD8A, CD8B, PRF1, GZMB for cytotoxic T cells; IL7R and LTB for CD4 T cell–enriched clusters; MKI67 and TOP2A for proliferating subsets; and FCER1A and CST3 for dendritic cell-enriched clusters. When reference-based labels and marker patterns were not fully concordant, clusters were assigned at the nearest higher level lineage category and were not used for fine-grained subgroup-specific interpretation. Differential expression and pathway enrichment analyses were conducted with Scanpy rank_genes_groups and clusterProfiler on GO and KEGG gene sets [27,33].

Cell–cell communication and lineage trajectories were inferred using CellPhoneDB and Monocle2, respectively. Because these methods are sensitive to sampling depth, cluster size, and group imbalance, and because our single-cell cohort includes five patients with a 4 to 1 responder imbalance, we interpret inferred interactions and trajectories as qualitative hypothesis-generating candidates rather than as calibrated between group differences that support confirmatory inference [34,35]. Motif analysis and chromatin accessibility deviations were derived from MACS2 peak sets, JASPAR 2020 motif annotations and chromVAR-based deviation scores [36,37,38]. TCR and BCR clonotypes were called with Cell Ranger VDJ. Only cells with productive paired TRA and TRB chains for TCR or productive paired heavy and light chains for BCR were retained, and unique paired chain combinations defined clonotypes. Clonal expansion and diversity indices were computed, and expanded clonotypes were mapped onto transcriptional states to explore associations between receptor usage and cytotoxic or exhausted phenotypes [8,36]. Because diversity and expansion estimates can be influenced by productive VDJ cell yield and effective sequencing depth, cross-sample comparisons were interpreted descriptively within this five patient cohort rather than as calibrated quantitative biomarkers. Given the limited number of patients, diversity metrics were interpreted descriptively, and emphasis was placed on concordant patterns across related indices rather than on formal hypothesis testing.

### 2.6. Statistical Analysis

Unless otherwise specified, analyses used two-sided tests with a significance threshold of adjusted *p* < 0.05 for high-dimensional comparisons such as bulk DEGs and enrichment analyses. For cell-level readouts, such as pathway scores or receptor expression, patient-level aggregation (for example, averaging within a given cell type per patient) was used for group comparison wherever possible to reduce pseudo-replication. Sensitivity analyses stratified by tissue type (primary tumor, lymph node, para-tumor) were performed for selected endpoints. For institutional single-cell figures that display error bars, variability was calculated across samples, meaning tissue libraries, within each response group and was reported descriptively. For the non-responder group, this variability reflected within patient cross-tissue variation rather than between patient variation.

For single-cell and multi-omic comparisons between responders and the single non-responder, *p*-values were treated as exploratory and were not considered confirmatory evidence given the very small sample size and potential for inter-patient variability. Throughout, effect sizes, consistency across related measurements and alignment with external datasets were prioritized over nominal statistical significance when interpreting the results. All analyses should therefore be viewed as hypothesis-generating and as providing a framework for future validation in larger independent cohorts.

## 3. Results

To provide context and maximize statistical robustness, we first analyzed bulk transcriptomic and immune-infiltration patterns in the larger GEO cohorts and then used the in-house multi-omic single-cell dataset as an exploratory resource.

### 3.1. Differential Transcriptomic and Immune Profiles Between TNBC and Non-TNBC in GSE76275

**Figure 1:**
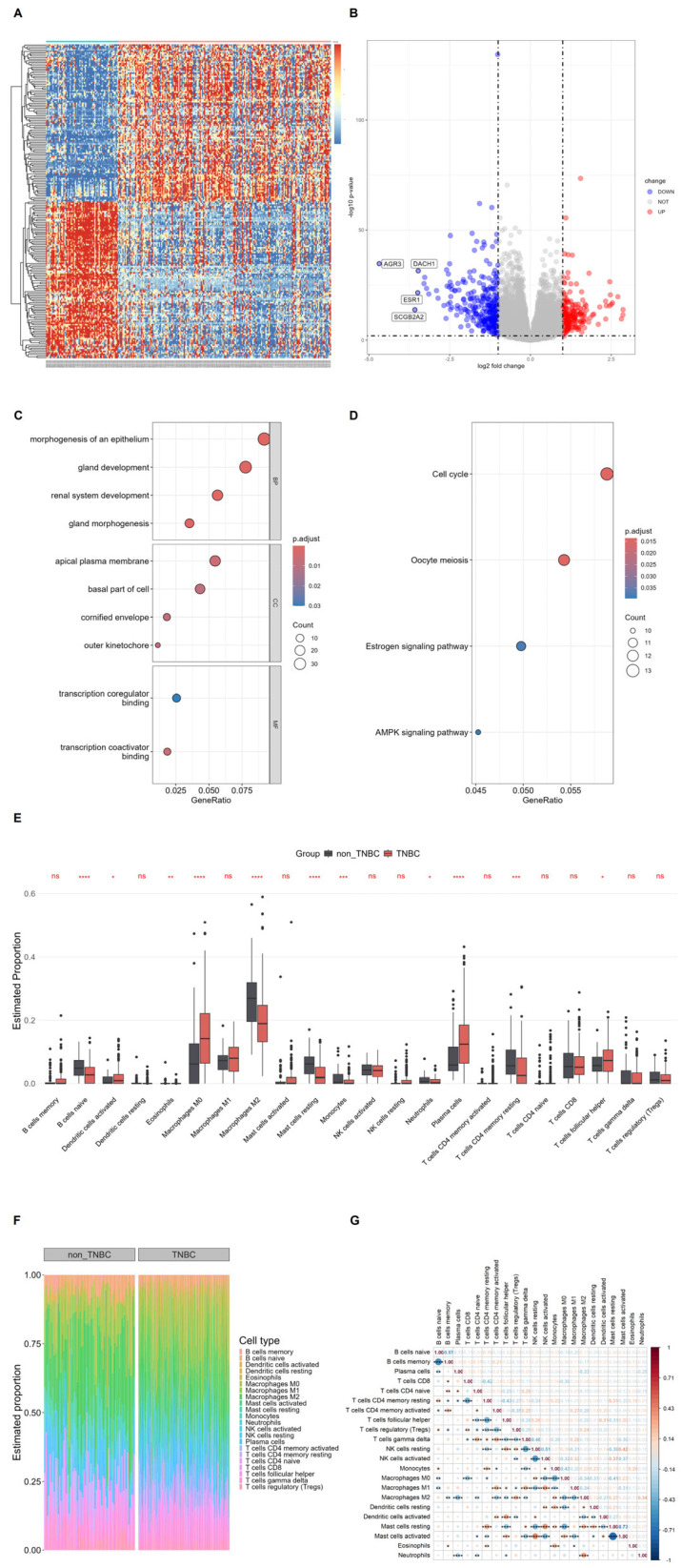
Transcriptomic and immune differences between TNBC and non-TNBC breast tumors in the GSE76275 cohort. (**A**) Heatmap of differentially expressed genes (DEGs; |log2FC| > 1, adjusted *p* < 0.05) separates TNBC from non-TNBC, showing distinct transcriptomic profiles. Rows = genes, columns = samples; red = high, blue = low expression. (**B**) Volcano plot of DEGs between TNBC and non-TNBC. Red = up-regulated in TNBC, blue = down-regulated, grey = non-significant. Representative luminal/ER-related genes (AGR3, DACH1, ESR1, SCGB2A2) are down-regulated in TNBC. Dashed lines mark fold-change (±1) and adjusted *p* = 0.05 thresholds. (**C**) GO enrichment of DEGs (BP, CC, MF). Key terms include epithelial morphogenesis, gland development, apical membrane, cornified envelope, and transcription co-regulator binding. Dot size = DEG count; color = adjusted *p*-value. (**D**) KEGG pathway enrichment highlights cell cycle, oocyte meiosis, estrogen signaling, and AMPK signaling, reflecting proliferative, hormonal, and metabolic differences. (**E**) Boxplots of 22 immune cell subsets (CIBERSORT) in TNBC (red) vs. non-TNBC (grey). Significance: ns (not significant), * (*p* < 0.05), ** (*p* < 0.01), *** (*p* < 0.001), **** (*p* < 0.0001). (**F**) Stacked bar plots of immune cell composition per tumor, showing heterogeneity across subtypes. (**G**) Correlation matrix of 22 immune cell subsets. Circle size/color indicate correlation strength/direction (red = positive, blue = negative), revealing coordinated B, T, and myeloid cell patterns.

In the GSE76275 cohort, TNBC and non-TNBC tumors displayed clearly distinct gene-expression and immune-infiltration patterns (Figure 1). Unsupervised clustering of DEGs (|log2FC| > 1, adjusted *p* < 0.05) separated samples into two major groups that aligned with TNBC and non-TNBC status, consistent with the presence of subtype-specific transcriptional signatures (Figure 1A). The volcano plot indicated that numerous genes were differentially expressed between TNBC and non-TNBC, with classical luminal/estrogen receptor-associated markers such as AGR3, DACH1, ESR1 and SCGB2A2 showing lower expression in TNBC (Figure 1B). Functional enrichment of DEGs highlighted biological processes related to epithelial morphogenesis and gland development, as well as cellular components such as the apical plasma membrane and cornified envelope and molecular functions involving transcription co-regulators (Figure 1C). KEGG analysis further suggested the differential involvement of cell-cycle and meiosis pathways together with estrogen and AMPK signaling (Figure 1D), underscoring differences in proliferative activity and hormone-dependent signaling between the two subtypes.

Immune deconvolution suggested that TNBC and non-TNBC tumors also differ in their immune microenvironment (Figure 1E–G). Compared with non-TNBC tumors, TNBC lesions tended to show higher estimated proportions of macrophages M0, neutrophils, plasma cells, eosinophils and T follicular helper cells, whereas non-TNBC tumors appeared enriched for naïve B cells, activated dendritic cells, macrophages M2, resting mast cells, monocytes and resting CD4^+^ memory T cells (Figure 1E). While not all comparisons remained significant after accounting for multiple testing, the overall pattern was reproducible across related cell types. Stacked bar plots illustrate substantial inter-tumor heterogeneity but broadly comparable immune complexity in both groups (Figure 1F), and correlation analysis reveals coordinated modules of B cell/T cell populations and myeloid subsets (Figure 1G). Together, these findings support a model in which TNBC is characterized by the loss of luminal ER transcriptional programs and, based on CIBERSORT deconvolution estimates, a relative enrichment of myeloid-associated immune fractions compared with non-TNBC breast tumors. Overall, these TNBC versus non-TNBC bulk results predominantly recapitulate established subtype biology and are presented here to validate the analytical workflow and to provide a robust reference context for interpreting the subsequent exploratory single-cell analyses.

### 3.2. Transcriptomic and Immune Correlates of NAC Response in TNBC Versus Non-TNBC

**Figure 2:**
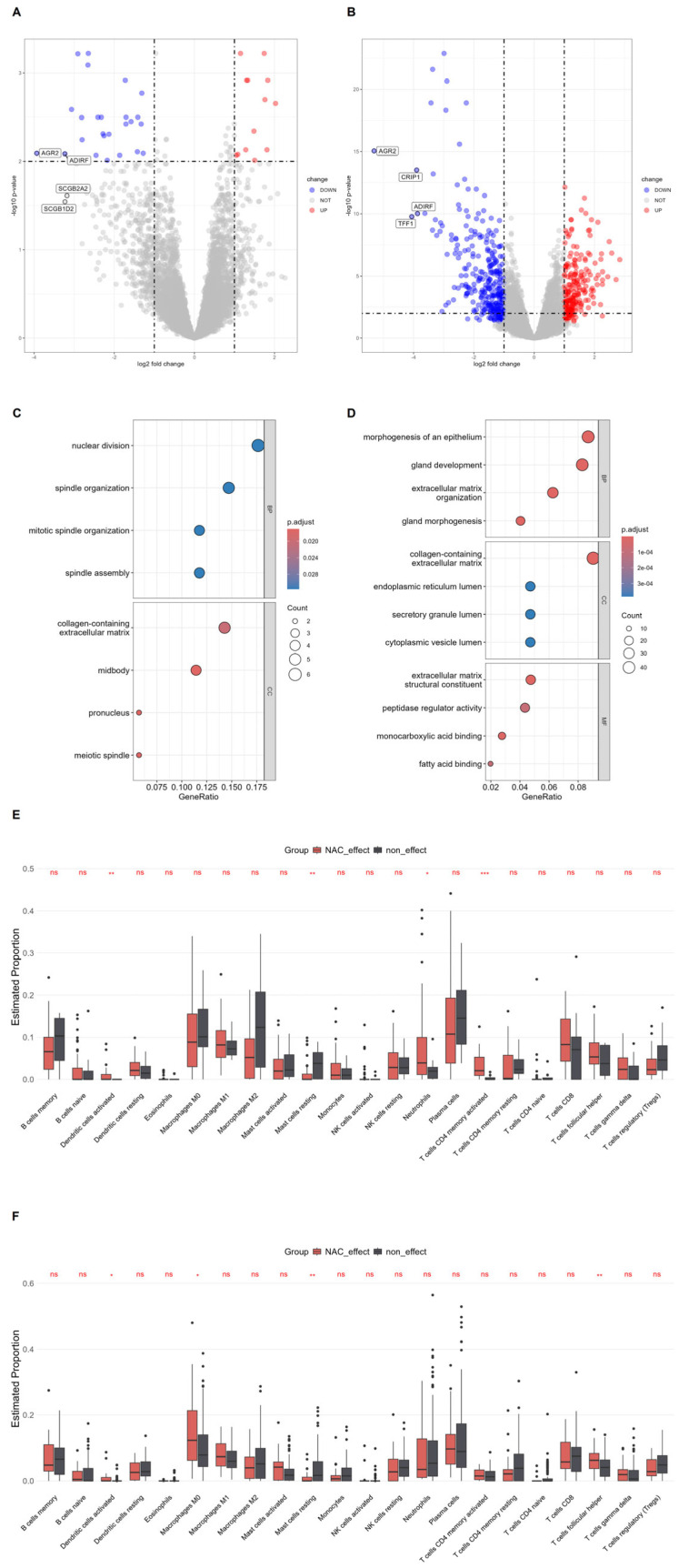
Differential transcriptomic and immune features associated with neoadjuvant chemotherapy response in patients with TNBC and non-TNBC from GSE25065. Panels in the left column (**A**,**C**,**E**) present analyses for the triple-negative breast cancer (TNBC) subgroup, while panels in the right column (**B**,**D**,**F**) show corresponding results for the non-TNBC subgroup. (**A**,**B**) Volcano plots of differentially expressed genes (DEGs) between NAC effect and non-effect tumors in patients with TNBC (**A**) and non-TNBC (**B**). The *x*-axis represents the log2 fold change, and the *y*-axis shows the –log10 *p*-value. Vertical dashed lines mark the fold-change thresholds, and the horizontal dashed line indicates the statistical significance cut-off. Genes with significant up- or down-regulation are highlighted, with representative examples labeled. (**C**,**D**) Gene Ontology (GO) enrichment analysis of DEGs in TNBC (**C**) and non-TNBC (**D**). Each dot corresponds to an enriched biological process, with dot size reflecting the number of DEGs involved and dot color indicating the adjusted *p*-value. The top enriched terms highlight pathways related to cell cycle, epithelial function, and stromal/secretory activity associated with NAC response in each subtype. (**E**,**F**) Estimated proportions of 22 tumor-infiltrating immune cell types in TNBC (**E**) and non-TNBC (**F**), comparing NAC effect (red) versus non-effect (grey) tumors using a CIBERSORT deconvolution method. Boxplots display the distribution of each immune cell subset, with statistical significance of group differences annotated as ns (not significant), * (*p* < 0.05), ** (*p* < 0.01), or *** (*p* < 0.001).

In GSE25065, we performed a responder versus non-responder comparison within the TNBC subset by contrasting pretreatment tumors from NAC responders (NAC effect) and non-responders (non-effect) (Figure 2). In TNBC (Figure 2A), there were only 38 DEGs, of which 25 were down-regulated and 13 were up-regulated, indicating that large, consistent transcriptional shifts associated with NAC response are not readily detectable at the bulk level in this subset. We emphasize that this low DEG count is itself a key negative result, suggesting that baseline bulk transcriptomes in TNBC may not yield robust single gene predictors of NAC response in unstratified cohorts and that response-associated biology may be distributed across cell states and microenvironmental compartments. NAC effect tumors showed reduced expression of several secretory and luminal-type genes, including AGR2, ADIRF, SCGB2A2 and SCGB1D2. GO enrichment of TNBC DEGs (Figure 2C) pointed to processes related to nuclear division, spindle organization and mitotic spindle organization, and cellular components such as the collagen-containing extracellular matrix and midbody, suggesting that subtle changes in proliferative activity and matrix architecture may accompany NAC response in TNBC, although effect sizes were modest.

By contrast, from the non-TNBC subset, we identified 553 DEGs with 321 down-regulated and 232 up-regulated genes. Here, NAC effect tumors again showed lower expression of secretory/luminal markers, including AGR2, ADIRF, CRIP1 and TFF1 (Figure 2B). Corresponding GO analysis (Figure 2D) indicated the enrichment of pathways related to morphogenesis of an epithelium, gland development and extracellular matrix organization, together with cellular component terms such as collagen-containing extracellular matrix, endoplasmic reticulum lumen and secretory granule lumen, and molecular functions linked to extracellular matrix structural constituents and peptidase regulation. These patterns suggest that, in non-TNBC, NAC response is accompanied by more extensive remodeling of epithelial and stromal programs than is evident in the TNBC subset.

Immune-cell deconvolution revealed only modest but biologically plausible shifts in the tumor immune microenvironment (Figure 2E,F). In TNBC (Figure 2E), non-effect tumors tended to harbor higher fractions of memory and naïve B cells, M0/M2 macrophages and resting mast cells, whereas NAC effect tumors showed relatively increased monocytes, neutrophils and activated CD4^+^ memory T cells but reduced resting NK cells. In non-TNBC (Figure 2F), NAC effect tumors exhibited a more clearly “inflamed” profile, with higher estimated proportions of macrophage subsets (M0–M2), neutrophils and CD8^+^ T cells, accompanied by lower levels of resting mast cells, plasma cells, naïve CD4^+^ T cells and regulatory/γδ T cell populations. Across immune subsets, immune differences associated with NAC response were modest and should be interpreted as small shifts in inferred immune composition rather than strong separations between responder and non-responder groups. Accordingly, we emphasize directionally consistent patterns across related immune subsets and treat nominal *p*-values as supportive rather than definitive evidence. These observations therefore point to combined moderate changes in tumor-intrinsic transcriptional programs and the composition of infiltrating immune cells, rather than a single dominant immune subset, as possible contributors to NAC response in this cohort. These modest bulk-level immune shifts in TNBC, particularly increased activated CD4^+^ memory T cells and myeloid inflammatory components in NAC effect tumors, align directionally with our exploratory single-cell cohort in which responders were enriched for T/NK cells and antigen-presenting mononuclear phagocytes.

### 3.3. Cell Composition and Immunological Heterogeneity in Responder and Non-Responder Tumors

**Figure 3:**
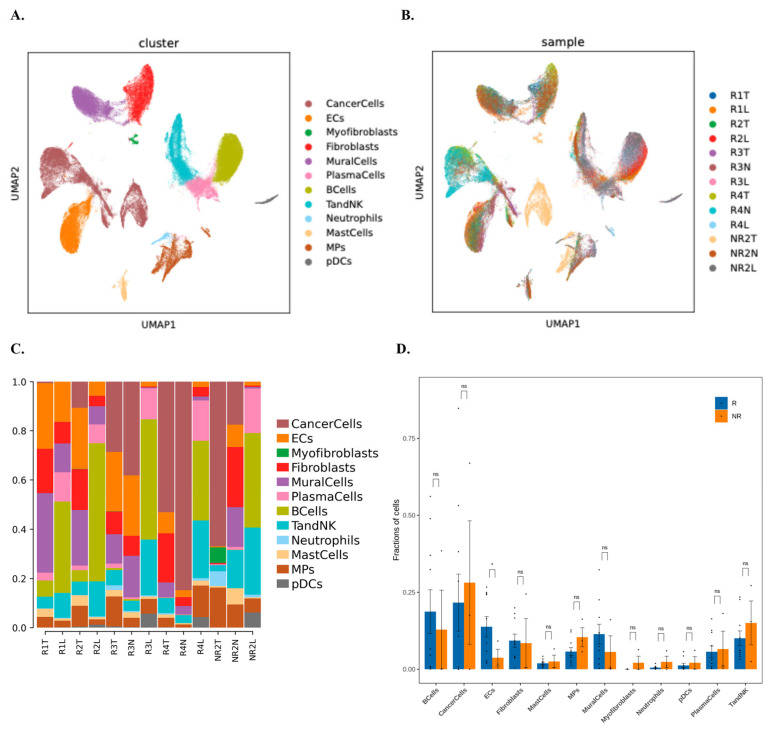
Cell composition of the tumor microenvironment across samples and response groups. (**A**) UMAP projection of all cells colored by cell type. Each dot represents a single cell and different colors indicate distinct cellular populations. (**B**) UMAP projection colored by sample. Each color corresponds to a different sample, illustrating the distribution and overlap of cells from individual patients. In panel B, T denotes primary tumor tissue, L denotes lymph node tissue, and N denotes para-tumor tissue. (**C**) Stacked bar plot showing the proportion of each cell type within each sample. The height of each colored segment represents the fraction of that cell type among all cells from the corresponding sample. (**D**) Bar plot summarizing the differential abundance of cell types between responder (R) and non-responder (NR) groups. The *x*-axis denotes cell types, and the *y*-axis shows the mean fraction of each cell type among total cells per sample in each group. Bars are colored by group (R vs. NR); error bars indicate the standard deviation across samples, meaning independently processed tissue libraries, within each group. Because the non-responder group includes one patient with multiple tissue samples, the non-responder error bars represent within-patient cross-tissue variability; asterisks mark exploratory differences. All panels derive from an exploratory single-cell cohort of five patients (four responders, one non-responder); between-group comparisons are descriptive and sensitive to sample imbalance and tissue-source heterogeneity. The ns” indicates no significant difference in the figure.

We next used our exploratory single-cell multi-omic dataset (n = 5; four responders and one non-responder) to explore how cellular composition of the tumor microenvironment might differ between NAC responders and the non-responder. Given the cohort size and sampling heterogeneity, downstream interpretation emphasizes lineage-level and recurrent ecosystem patterns that are robust to modest uncertainty in fine-grained subcluster boundaries. In Figure 3, UMAP clustering analysis revealed 12 major cell populations, including cancer epithelial cells, fibroblasts, endothelial cells, mononuclear phagocytes (MPs), T and NK cells, B cells and neutrophils. Both responder (R) and non-responder (NR) tumors contained these core populations but their relative abundance varied.

In this small cohort, responder tumors tended to exhibit higher proportions of tumor-infiltrating lymphocytes (T/NK cells), MPs and dendritic cells, consistent with an immune-activated microenvironment, whereas the non-responder sample displayed relatively higher fractions of cancer cells, fibroblasts and mast cells, suggestive of a more stromal- and tumor-dominated milieu. Quantitative summaries in Figure 3D reflect mean cell-type fractions per sample within each response group. In this dataset, the NR group shows a higher mean T and NK fraction, while responders show lower mean values for this compartment in the same summary. Given that the NR group comprises a single patient and samples include multiple tissue sources, these differences should be interpreted descriptively as cohort limited patterns rather than as definitive response-associated composition shifts.

### 3.4. Differential Gene Expression Suggests Immune Activation in Responders and Secretory/Cell-Adhesion Programs in the Non-Responder

**Figure 4:**
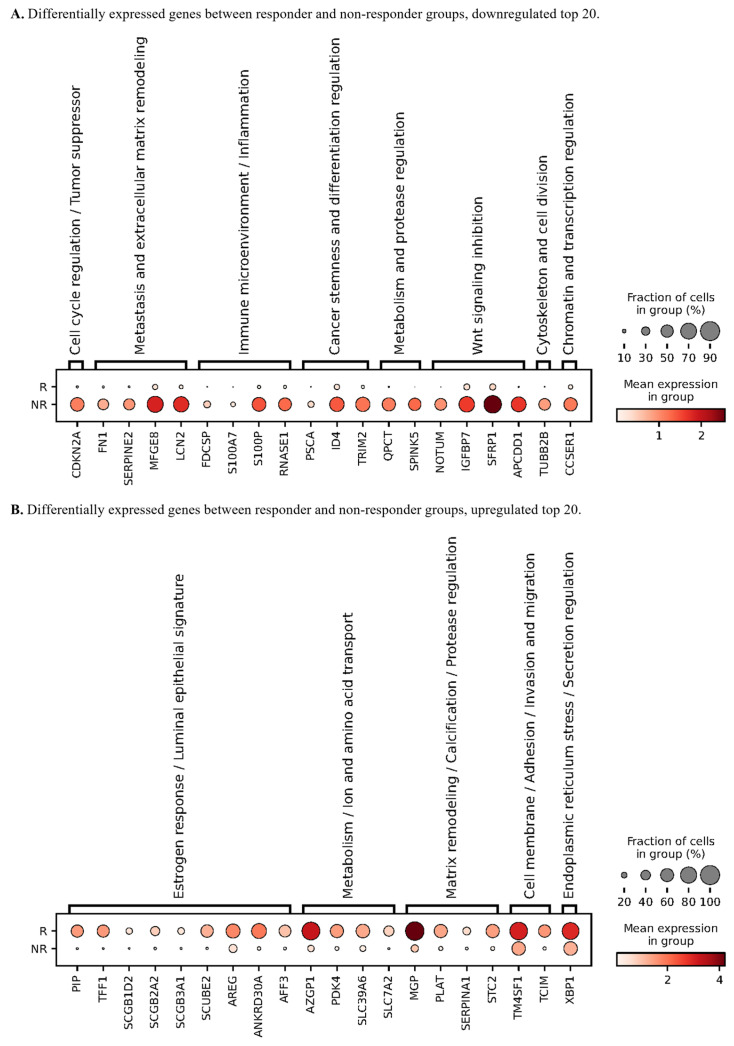
Differentially expressed genes between responder and non-responder groups. (**A**) Bubble plot showing the top 20 differentially expressed genes (DEGs) that are down-regulated in the responder (R) group compared with the non-responder (NR) group. (**B**) Bubble plot showing the top 20 DEGs that are up-regulated in the non-responder (NR) group compared with the responder (R) group. All panels derive from an exploratory single-cell cohort of five patients (four responders, one non-responder); between-group comparisons are descriptive and sensitive to sample imbalance and tissue-source heterogeneity.

In this exploratory cohort of five patients (four responders and one non-responder), single-cell differential expression analysis was performed to explore transcriptional programs that differed between response groups across the sampled tumor ecosystem. Responder up-regulated genes were dominated by epithelial secretory and differentiation associated transcripts, including PIP, TFF1, AZGP1, SCGB1D2, SCGB2A2, SCUBE2, ANKRD30A, AREG, XBP1, STC2, and SLC39A6 (Figure 4A), with higher expression observed in both scaled average expression and the fraction of expressing cells in the responder group. In contrast, the non-responder showed a higher expression of genes consistent with extracellular matrix interaction, adhesion and remodeling, and epithelial stress or inflammatory programs, including FN1, SERPINE2, IGFBP7, RNASE1, SFRP1, NOTUM, MFGE8, S100A7, LCN2, S100P, SPINK5, APCDD1, and CDKN2A (Figure 4B). Collectively, within this dataset, responders were characterized by a more prominent secretory or differentiated epithelial program, whereas the non-responder was characterized by ECM-associated and stress-linked transcriptional features. Because these comparisons contrast four patients with one and are sensitive to inter-individual and sample-source heterogeneity, we present these DEGs as candidate programs for future validation rather than as definitive responder- or non-responder-specific signatures.

### 3.5. TCR/BCR Clonotype Patterns and Adaptive Immune Potential

**Figure 5:**
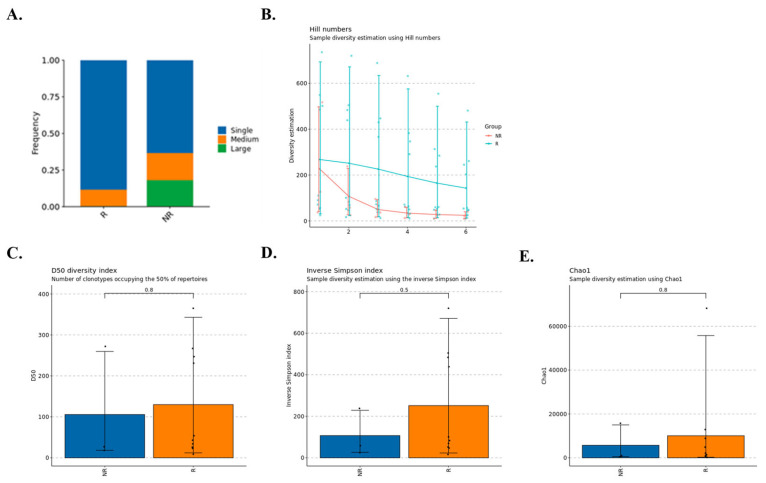
TCR/BCR clonotype diversity and expansion across responder and non-responder groups. Repertoire diversity and expansion metrics may be influenced by productive VDJ cell counts and effective sampling depth, therefore responder versus non-responder comparisons are descriptive within this exploratory cohort. (**A**) Bar plot showing the distribution of TCR/BCR clonotype expansion levels (single, medium, and large) in responder (R) and non-responder (NR) groups. The height of each colored segment indicates the proportion of clonotypes within each expansion category, illustrating differences in clonotype expansion patterns between groups. (**B**) Hill numbers-based diversity analysis comparing R and NR groups, showing decreasing diversity estimates as parameter q increases. The figure highlights lower diversity in highly expanded clonotypes, particularly in the NR group. Error bars represent variability across samples. (**C**) Boxplot of the D50 diversity index, representing the number of unique clonotypes that make up 50% of the total repertoire. Higher D50 values indicate greater clonotype diversity. (**D**) Boxplot of the inverse Simpson index, showing overall repertoire diversity. Higher index values indicate more even and diverse clonotype distribution. (**E**) Boxplot of the Chao1 diversity estimator, measuring repertoire richness based on rare clonotypes. Values represent estimated total clonotype richness, with error bars reflecting sample-level variability. All panels derive from an exploratory single-cell cohort of five patients (four responders, one non-responder); between-group comparisons are descriptive and sensitive to sample imbalance and tissue-source heterogeneity. Error bars indicate the standard deviation across samples, meaning independently processed tissue libraries, within each group. Because the non-responder group includes one patient with multiple tissue samples, the non-responder error bars represent within-patient cross-tissue variability.

In this exploratory five-patient cohort, we examined TCR/BCR clonotype architecture as a proxy for adaptive immune engagement (Figure 5). Responder tumors appeared to harbor a greater proportion of medium to large clonotype clusters, indicative of active clonal expansion and antigen-driven immune responses, whereas the non-responder sample displayed fewer expanded clones. Diversity metrics, including Hill numbers, D50, inverse Simpson and Chao1 indices, generally trended toward higher repertoire richness and evenness in responder samples, suggesting a broader diversification of immune clones in association with NAC response. These repertoire metrics are interpreted as correlates of adaptive immune engagement and do not by themselves establish tumor antigen specificity, effector function, or a causal role in tumor clearance in the absence of functional validation.

### 3.6. Glycosylation Remodeling and Immune-Associated Glyco-Signaling in Responder Tumors

**Figure 6:**
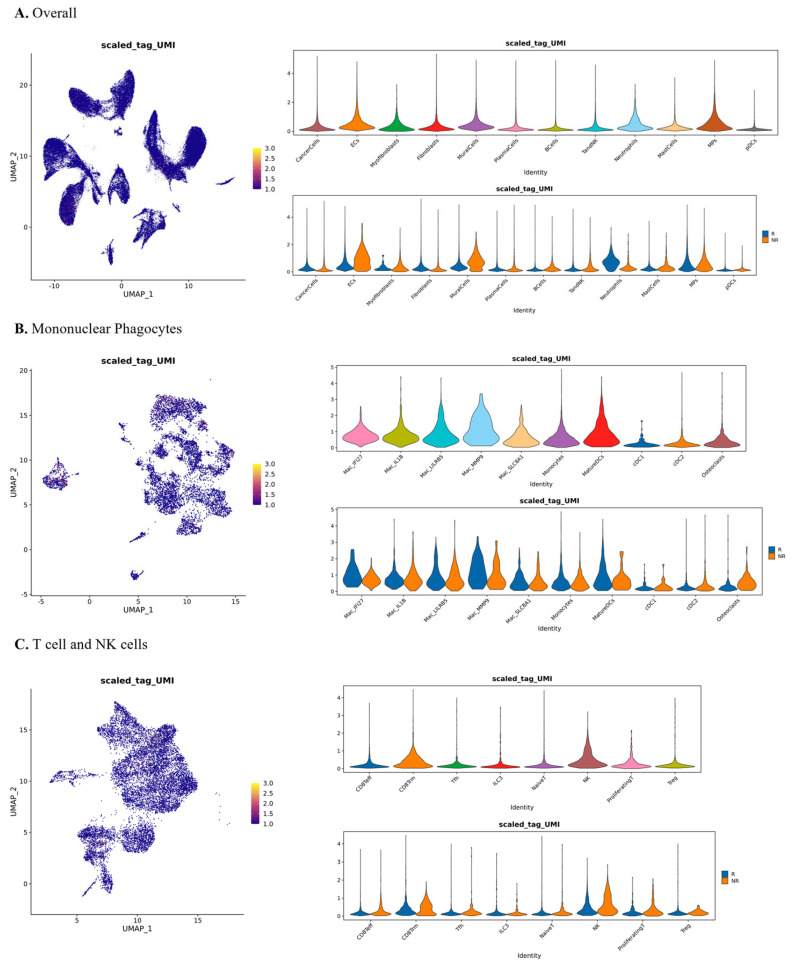
Cell type-specific glycosylation profiles across responder and non-responder groups. Scaled_tag_UMI denotes the scaled ProMoSCOPE glyco-tag counts per cell, used here as a proxy for relative cell-surface glycan-tag signal. (**A**) Overall glycosylation signal across all major cell types, showing both cell type-specific variation and differences between responder (R) and non-responder (NR) groups. (**B**) Glycosylation patterns within mononuclear phagocytes (MPs), comparing glyco-tag expression across MP subsets and between R and NR groups. (**C**) Glycosylation signal in T cell and NK cell populations, illustrating subgroup-specific glycosylation differences and treatment response-associated patterns. All panels derive from an exploratory single-cell cohort of five patients (four responders, one non-responder); between-group comparisons are descriptive and sensitive to sample imbalance and tissue-source heterogeneity.

In this exploratory five-patient cohort, glycosylation profiling provided an additional layer of information on cell-surface glyco-signaling (Figure 6). Cell-surface glycans can modulate protein–protein interactions by influencing receptor folding and stability, trafficking and surface abundance, and the steric or conformational accessibility of binding interfaces; glycan motifs can also create or mask interaction sites for lectins and immune receptors. We therefore interpret glyco-tag intensity as a readout of glycan remodeling with the potential to tune receptor–ligand engagement between immune and tumor cells. At the global level, responders tended to exhibit elevated glyco-tag signals in immune-active compartments, including MPs, T cells, NK cells and dendritic cells, whereas the non-responder sample showed glycosylation signals more confined to tumor epithelial and stromal cells. In mononuclear phagocytes, responders showed higher glyco-tag intensity in macrophages and conventional dendritic cells (cDCs), consistent with a microenvironment in which antigen-presenting cells exhibit greater surface glycan remodeling. In T and NK subpopulations, responders displayed stronger glycosylation enrichment in effector memory CD8^+^ T cells and cytotoxic NK subsets, consistent with the possibility that glycan remodeling could modulate immune synapse receptor–ligand interactions in these cytotoxic compartments.

### 3.7. Checkpoint Ligand–Receptor Communication and Candidate Immunoregulatory Pathways

**Figure 7:**
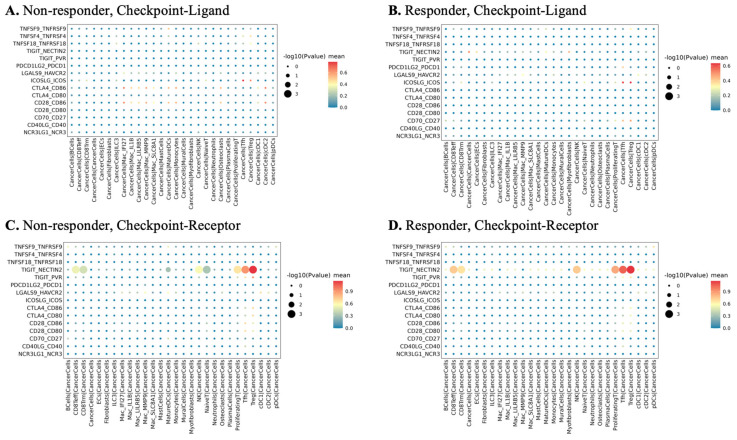
Expression patterns of checkpoint ligands and receptors in responder and non-responder groups. (**A**) Checkpoint ligand expression across cell–cell pairs in the non-responder (NR) group. (**B**) Checkpoint ligand expression across cell–cell pairs in the responder (R) group. (**C**) Checkpoint receptor expression across cell–cell pairs in the non-responder (NR) group. (**D**) Checkpoint receptor expression across cell–cell pairs in the responder (R) group. All panels derive from an exploratory single-cell cohort of five patients (four responders, one non-responder); between-group comparisons are descriptive and sensitive to sample imbalance and tissue-source heterogeneity.

Given the strong sample size imbalance and dependence of ligand receptor inference on cell number and expression detection, the following results are presented as candidate interaction patterns that require validation in larger balanced cohorts. In this exploratory five-patient cohort, CellPhoneDB-based cell–cell communication analysis was used to nominate candidate checkpoint-related ligand–receptor pairs based on cell type-specific co-expression patterns in this dataset (Figure 7). In the non-responder sample, checkpoint signaling appeared relatively limited, with low predicted ligand–receptor activity across cell types. By contrast, responders showed higher TIGIT expression in proliferating CD8^+^ T and NK subsets and higher NECTIN2 expression in mononuclear phagocytes, and CellPhoneDB nominated TIGIT–NECTIN2 as a candidate ligand–receptor pairing based on co-expression patterns. The other classical checkpoints (PDCD1, CTLA4, ICOS and TNFRSF members) showed low or modest expression in both groups. Within the constraints of this small cohort, these findings support an expression and predicted pairing signal for TIGIT–NECTIN2 but do not establish physical interaction, pathway activation, or functional checkpoint activity. We view this axis as a candidate target for further investigation rather than as the sole or predominant determinant of response.

## 4. Discussion

In this work we combined public bulk cohorts with a small in-house single-cell multi-omic dataset to explore how tumor intrinsic programs, immune composition, receptor repertoires and glycosylation features relate to neoadjuvant chemotherapy response in triple-negative breast cancer. The bulk analyses reproduced established differences between triple-negative and non-triple-negative disease and between responders and non-responders, supporting the internal validity of the dataset and anchoring our single-cell observations in a broader clinical context. Accordingly, we frame the bulk subtype contrasts primarily as validation and contextualization rather than as a primary novelty contribution. Many analyses have shown that pathologic complete response after neoadjuvant chemotherapy is strongly associated with improved survival in triple-negative breast cancer, although many patients do not achieve complete clearance of invasive disease [4,39]. Our bulk data confirm that triple-negative tumors are enriched for proliferation-associated programs and have a more myeloid-oriented immune infiltrate relative to non-triple-negative tumors, which is consistent with prior subtype-focused transcriptomic studies.

The single-cell multi-omic profiles extend these observations by indicating that baseline ecosystems associated with NAC response differ along both immune and tumor-intrinsic axes. Cell type fractions varied across samples and tissue sources, and Figure 3D summarizes mean fractions per sample. In this per sample summary, the non-responder group, representing a single patient in our five-patient cohort with four responders and one non-responder, shows a higher mean T and NK fraction, while responders show a higher representation of antigen-presenting mononuclear phagocytes and dendritic cells. Therefore, we interpret these composition differences descriptively and as hypothesis-generating patterns within an imbalanced cohort rather than as definitive response-associated shifts. The single-cell multi-omic profiles refine this context by suggesting that baseline ecosystems associated with neoadjuvant response vary along two coupled dimensions, an immune engagement dimension and a tumor stromal dominance dimension. Rather than attributing response to a single-cell type shift, we emphasize the cross-modality concordance that emerges when cellular composition, receptor repertoire architecture, glycosylation signals, and inferred checkpoint programs are considered together. In responders, antigen presentation-related myeloid compartments, and cytotoxic lymphocytes co-occur with adaptive repertoire activation patterns, immune-associated glycosylation, and inducible checkpoint signaling, consistent with a microenvironment capable of mounting and regulating adaptive immune interactions during therapy. In the non-responder, epithelial and stromal programs are comparatively dominant, together with extracellular matrix, adhesion, and stress-associated transcriptional states, which may constrain immune niche formation or effector access. Because the cohort is small and imbalanced, these contrasts are interpreted descriptively and as hypothesis-generating patterns rather than as definitive response- associated shifts. On the immune axis, responders show features consistent with active immune engagement across orthogonal readouts, including adaptive receptor repertoire patterns, immune-associated glycosylation signals, and checkpoint communication programs. On the tumor intrinsic axis, the differential expression patterns in Figure 4 are dominated by epithelial state programs rather than by a uniform immune activation signature [13,14,40,41]. The responder-enriched DEGs primarily reflect secretory or differentiation-associated epithelial transcription, whereas the non-responder is characterized by extracellular matrix or adhesion-associated and stress-linked transcriptional features [13,40,41]. Given that the non-responder group comprises a single patient, these contrasts should be interpreted descriptively.

The T cell and B cell receptor analyses provide an additional layer of evidence for adaptive immune engagement. Prospective studies in breast cancer have shown that circulating T cell receptor repertoires become more clonally expanded and that higher clonal expansion and diversity indices are associated with better neoadjuvant chemotherapy response and with higher tumor mutation burden [42,43]. Reviews of T cell receptor repertoire profiling in solid tumors highlight that a diverse yet clonally expanded repertoire is often linked to effective antitumor immunity, whereas restricted or oligoclonal patterns may signal immune escape [44]. In our dataset, responders tended to show more medium and larger clonotype clusters and higher diversity metrics than the non-responder, which is directionally consistent with these reports. However, repertoire diversity and clonal expansion are correlative indicators of antigen experience and immune recruitment and do not by themselves establish tumor antigen specificity, cytotoxic activity, or a causal role in tumor clearance; accordingly, these findings should not be equated with effective antitumor immunity in the absence of functional validation. Given the very small sample size these results should be interpreted as qualitative support for a link between adaptive immune breadth and chemotherapy sensitivity rather than as a calibrated biomarker proposal.

Glycosylation profiling in single cells suggests that immune related glycol-signaling may differ between responders and non-responders. Experimental work has established that N-linked glycosylation of PD-L1 stabilizes the protein, preserves its interaction with PD-1 and promotes immune evasion in triple-negative breast cancer models [45,46]. More recent studies in human tumors have implicated broader glycosylation programs and lectin-like factors as modulators of antigen presentation, interferon signaling and immune infiltration in triple-negative disease [47]. Clinical series have also reported that deglycosylated PD-L1 can sharpen the association between PD-L1 protein levels and response to immune checkpoint blockades [48]. Within this framework, our observation that responders exhibit stronger glycol-tag signals in mononuclear phagocytes and cytotoxic lymphocytes whereas the non-responder shows glycosylation largely confined to epithelial and stromal compartments, is compatible with a model in which glycan remodeling in immune cells contributes to effective antigen presentation and effector function. By contrast, tumor-centered glycosylation in the absence of matching immune glycol remodeling may reflect immune evasion programs that persist despite cytotoxic therapy.

The analysis of checkpoint ligand–receptor communication highlights TIGIT and its ligand CD155 as a candidate pathway in neoadjuvant chemotherapy response. Preclinical work has shown that CD155 TIGIT interactions dampen cytotoxic T cell activity and promote immune escape in triple-negative breast cancer [49]. In patients with residual triple-negative disease after neoadjuvant chemotherapy high CD155 expression associates with adverse outcome, whereas CD73 shows a different pattern, which underscores the complexity of purinergic and nectin family signaling in this context [50]. A recent clinical study further reported that a composite score integrating CD155, TIGIT and related receptors on tumor and stromal cells can enrich neoadjuvant chemotherapy-resistant triple-negative tumors [51]. In our cohort TIGIT and NECTIN2 expression and inferred communication were more prominent in responders than in the non-responder. This pattern is compatible with a state of active antitumor immunity with inducible but potentially reversible checkpoint engagement rather than with complete immune exclusion. Together with existing data it suggests that CD155 TIGIT signaling should be evaluated not only as a resistance pathway but also as a context dependent marker of ongoing immune surveillance in chemotherapy-treated triple-negative breast cancer.

This study has important limitations. The single-cell and multi-omic analyses rely on only five patients, with a strong imbalance between responders and the non-responder and are therefore underpowered for formal hypothesis testing. All observations from the single-center cohort should be regarded as exploratory and require validation in larger and more diverse populations. Technical choices, including dissociation protocols, platform-specific chemistry and bioinformatic thresholds, may also influence the detection of rare states and the quantification of clonotypes and glycol-tags. In addition, we did not perform spatial validation such as spatial transcriptomics or multiplex imaging. As a result, we cannot confirm the tissue-level organization of immune and stromal niches, the proximity of cytotoxic lymphocytes to antigen-presenting myeloid compartments, or the spatial plausibility of inferred ligand–receptor communication. Spatially resolved follow-up studies will be needed to test whether the proposed ecosystems reflect coordinated microenvironmental architectures associated with chemotherapy response. Nonetheless, the concordance between our bulk transcriptomic and immune deconvolution findings and prior large-scale work, together with the directional agreement between our single-cell patterns and published single-cell and spatial studies in triple-negative breast cancer, support the biological plausibility of the proposed models [3,4,5,6,8,9,10,11,12,13,14]. Future studies that integrate single-cell transcriptomics, spatial profiling, T cell and B cell receptor sequencing and targeted glycoproteomics in larger neoadjuvant chemotherapy cohorts will be needed to determine whether combined descriptors of cellular composition, receptor architecture, glycosylation and checkpoint signaling can refine patient stratification beyond current clinicopathologic and bulk genomic markers.

## 5. Conclusions

In this study we integrated public bulk gene expression cohorts with a small, deeply profiled single-cell and multi-omic dataset to explore determinants of neoadjuvant chemotherapy response in triple-negative breast cancer. Bulk transcriptomic and immune deconvolution analyses recapitulated established differences between triple-negative and non-triple-negative disease and suggested that chemotherapy response is associated with coordinated but moderate changes in tumor intrinsic programs and immune composition rather than a single dominant pathway. However, we must fully acknowledge and emphasize the limitation of the single-cell cohort, in which the sample size is small and unbalanced. Specifically, the comparative group consisted of five triple-negative breast cancers (n = 5), including four responders (n = 4) and only one non-responder (n = 1). This restricted sample size, particularly the single non-responder, inevitably limits the strength of our comparative conclusions.

The single-cell multi-omic analyses provide a detailed description of cellular states in this small single-center series. The four pathologic complete response (pCR) tumors showed immune-enriched microenvironments with higher proportions of T and natural killer cells, antigen-presenting myeloid populations, broader T and B cell receptor repertoires, and immune-associated glycosylation and checkpoint features. The single non-pCR tumor was dominated by epithelial and fibroblast compartments with secretory and adhesion programs and comparatively restricted adaptive immune features. Because the non-pCR group comprised only one patient and triple-negative breast cancer is highly heterogeneous, these observations cannot support between-group inference and the non-pCR sample may represent an outlier. Accordingly, the single-cell results should be regarded as descriptive and as proof of concept for integrated bulk and single-cell multi-omic profiling. The reported patterns require validation in larger independent cohorts, ideally with spatially resolved assays, and should not be used for patient stratification or treatment decisions without such validation.

## Data Availability

Transcriptional data used in this study were obtained from publicly accessible Gene Expression Omnibus (GEO) datasets. The in-house human sample data can be made available for reasonable research purposes upon discussion with, and approval from, the institutional ethics committee, in accordance with ethical considerations.

## Abbreviations

The following abbreviations are used in this manuscript:

TNBC	Triple-negative breast cancer
NAC	Neoadjuvant chemotherapy
GEO	Gene Expression Omnibus
DEGs	Differentially expressed genes
FC	Fold change
FDR	False discovery rate
GO	Gene Ontology
KEGG	Kyoto Encyclopedia of Genes and Genomes
